# The relationship between occupational stress and job burnout among female manufacturing workers in Guangdong, China: a cross-sectional study

**DOI:** 10.1038/s41598-022-24491-0

**Published:** 2022-11-23

**Authors:** Shanyu Zhou, Huiqing Chen, Ming Liu, Tianjian Wang, Haijuan Xu, Rongzong Li, Shibiao Su

**Affiliations:** Guangdong Province Hospital for Occupational Diseases Prevention and Treatment, Guangdong Provincial Key Laboratory of Occupational Disease Prevention and Treatment, No.68 Haikang Street Xingang Road West, Guangzhou, 510300 China

**Keywords:** Health occupations, Risk factors

## Abstract

This study aims to investigate the relationship between occupational stress and job burnout in female manufacturing workers. A random sample of 1081 female workers in electronic manufacturing in Guangdong Province participated in the present study. An anonymous self-administered questionnaire that covered social-demographic characteristics, the Chinese version of the Job Content Questionnaire, the Chinese version of the Effort-reward Imbalance Questionnaire, and the Maslach Burnout Inventory for the General Survey, was used to assess occupational stress and job burnout. Independent sample t-test, one-way analysis of variance (ANOVA), correlation analysis, hierarchical multiple regression analysis and logistic regression analysis were used in data analysis. Occupational stress was positively correlated with emotional exhaustion and depersonalization and negatively correlated with personal accomplishment. After adjusting for sociodemographic characteristics, job strain was a risk factor for emotional exhaustion (*OR* = 2.27, 95% *CI*: 1.61–3.20) and depersonalization (*OR* = 1.96 *95% CI*: 1.45–2.64). Female workers with high effort-reward imbalance had an increased risk of depersonalization (*OR* = 1.96, *95% CI*: 1.33–2.90). Furthermore, female workers with high overcommitment had an increased risk of emotional exhaustion (*OR* = 3.07, 95% *CI*: 2.06–4.58) and depersonalization (*OR* = 2.83, 95% *CI*: 1.92–4.17), while higher social support reduced the risk of emotional exhaustion (*OR* = 0.37, 95% *CI*: 0.26–0.53). The job burnout of female manufacturing workers is significantly correlated with their occupational stress. Higher job strain and overcommitment might be important contributors to job burnout. Increased worker social support can reduce job burnout.

## Introduction

Stress has been considered a psychological problem that seriously affects human beings in modern society^1^. Stress in the workplace is inevitable due to the demands of the contemporary work environment. Based on a survey, 40% of workers mentioned that their job was very or extremely stressful, 26% of workers were often burned out or stressed by their work, and 29% of workers were quite slightly or extremely stressed at work^2,3^. Acceptable occupational stress may keep workers alert, motivated and hard-working and allow them to learn. However, when stress becomes excessive or otherwise unmanageable, it damages the health and performance of employees. Stress has been assumed to be a trigger that causes adverse conditions and deteriorates health. The levels of stress-related illnesses have been nearly twice as high for women when compared to men^4^. Epidemiological studies have found that female workers with stressful jobs have an increased risk of cardiovascular disease, hypertension, musculoskeletal diseases, depression, poor mental health and adverse health behaviors, such as alcoholism, smoking, physical inactivity and obesity^5–11^.

Burnout is a complex phenomenon that involves individual experiences that interact with the social and environmental contexts of the workplace^12^. Maslach described burnout as a multidimensional condition that comprises emotional exhaustion, depersonalization and reduced personal accomplishment. In addition, he further described that burnout begins when individuals are overwhelmed with the unexpected and unbearable stressful aspects of the job, which frustrates their efforts to make a passive impact on others^13^. Burnout is associated with turnover intention, lower productivity, and decreased commitment^14–16^. Research has shown that job burnout is a combination of emotional exhaustion, depersonalization and low personal accomplishment due to long periods of occupational stress^17^.

In China, the relationship between occupational stress and job burnout has been studied among doctors and teachers^18,19^. The monotony of assembly line work can cause occupational stress in employees, but there are few studies on the occupational stress of such female manufacturing workers and occupational burnout. Since female workers are the main work force of China’s wide range of manufacturing sectors, they are at risk of exposure to hazardous chemicals, physical demands, noisy equipment, and long work hours. With more jobs shifting from the manufacturing sector to the service sector, they also face the challenge of learning new technologies and knowledge, which would increase their work pressure. Therefore, studying the relationship between occupational stress and job burnout is of great significance to identify the main stresses and design intervention measures to reduce stress-related job burnout.

## Materials and methods

### Setting and participants

This cross-sectional study was conducted in 5 large electronic manufacturing companies in Zhuhai, Foshan, Zhongshan, Shenzhen and Dongguan cities of Guangdong Province. The basic characteristics of the 5 companies are presented in Table S1. The method of multistage stratified cluster random sampling was adopted to acquire the sample. First, 5 sample districts from the Pearl River Delta of Guangdong Province were randomly selected. Second, 5 electronic manufacturing companies were chosen from each sample district. All female workers from 5 companies were selected. To facilitate the data collection, all female employees (*N* = 1200) were selected as participants and were invited to complete an anonymous questionnaire. A total of 1081 female workers volunteered to complete the questionnaire. The response rate was 90.0% (1081/1200). The present study was approved by the ethics committee of Guangdong Province Hospital for Occupational Diseases Prevention and Treatment and was strictly compliant with local law and the Declaration of Helsinki. All participants provided informed consent prior to administering the survey. All methods were performed in accordance with relevant guidelines and regulations.

### Data collection

The data collection was carried out in 2016 using an anonymous self-administered questionnaire. The questionnaire covered sociodemographic characteristics (age, marital status, educational level, monthly income, shift work, assembly line, working years and exposure to occupational hazards) and the question measures of occupational stress and job burnout.

### Measurement of occupational stress

Occupational stress was assessed through two well-known questionnaires: Karasek’s Job Content Questionnaire (JCQ) and the Effort Reward Imbalance Questionnaire (ERIQ)^20,21^. The Chinese versions of these two questionnaires both demonstrated good reliability and validity in Chinese populations^22,23^.

The Chinese version of the 22-item JCQ included three components: job demand (five items), job control (nine items) and social support (eight items). Each of these items was rated using a 4-point Likert-type scale, varying from 1 (strongly disagree) to 4 (strongly agree). The Job Demand-control Model defines the work environment with high job demands and considers little job control as job strain. The ratio score of the job strain was calculated based on the “job demand score”/“job control score” (weighted by item numbers). A higher ratio score indicates higher occupational stress. The ratio was divided into three parts based on the tertiles. Subjects in the upper tertiles of the demand-control ratio were defined as the high job strain group, those in the middle tertiles were defined as the moderate job strain group, and those in the lower tertiles were defined as the low job strain group. In the present study, Cronbach’s alpha coefficients of job demand, job control and social support were 0.698, 0.729 and 0.882, respectively.

The ERIQ was built on the effort-reward imbalance model, which consists of three dimensions: extrinsic effort (six items), reward (eleven items), and overcommitment (six items). The items of extrinsic effort and reward were rated using a 5-point scale, which ranged from 1 to 5. The items of effort and reward are answered in two steps. First, the participants agreed or disagreed with whether the content of the items described a typical experience in their work situation. If they agreed with it, they were asked to evaluate to what extent they usually felt distressed by this typical experience. The items of overcommitment were rated using a 4-point scale, which varied from 1 to 4. The effort-reward imbalance model was based on social reciprocity. Failed social reciprocity at work elicits occupational stress^24^. The ratio of extrinsic effort sum scores and reward sum scores (weighted by item numbers) was calculated to quantify the degree of effort-reward imbalance. These ratios were divided into three parts based on the tertiles. Subjects in the upper tertiles of the effort-reward ratio were defined as the high effort-reward imbalance group, those in the middle tertiles were defined as the moderate effort-reward imbalance group, and those in the lower tertiles were defined as the low job effort-reward imbalance group. In the present study, Cronbach’s alpha coefficients of job demand, job control and social support were 0.652, 0.850 and 0.796, respectively.

### Measurement of job burnout

Job burnout was assessed through the Maslach Burnout Inventory-General Survey (MBI-GS)^25^, which consists of three dimensions: emotional exhaustion (five items), depersonalization (four items) and personal accomplishment (six items). The Chinese version of the MBI-GS demonstrated good reliability and validity in the Chinese population^18,19^. All items were valued using a 7-point scale, which ranged from 0 to 6 (“never” to “every day”). The score of each dimension was separately computed. Emotional exhaustion and depersonalization scores of > 66.7% and personal accomplishment scores of < 33.3% were defined as low and high scores, respectively. People in the high group of emotional exhaustion and depersonalization, together with those in the low group of personal accomplishment, were defined as suffering job burnout. In the present study, Cronbach’s alpha coefficients of emotional exhaustion, depersonalization and professional accomplishment were 0.872, 0.774 and 0.852, respectively.

### Statistical analysis

All statistical tests were two-sided (α = 0.05). Independent sample *t* test or one-way analysis of variance (ANOVA) was used to compare the means of scores in the different sociodemographic groups. *Pearson’s* correlation coefficients were calculated to examine the correlation among the study variables of the MBI-GS, JCQ and ERIQ. Hierarchical linear regression analyses were conducted to examine the association among the JCQ, ERIQ and MBI-GS scores. The control variables were added into the model at the first step of the hierarchical linear regression analysis. In the present study, sociodemographics, including age, marital status, educational level, monthly income, shift work, assembly line, working years and exposure to occupational hazards, were set as confounders. Dummy variables were set for marital status, educational level, monthly income and working years. Furthermore, “married”, “junior school or less”, “ < ¥1500”, and working years “ < 2” were set as the reference groups. The dimensions of the JCQ and ERIQ were added in step 2. The variances of the MBI-GS scores explained by occupational stress were examined by ∆*R*^2^. A nonconditional logistic regression model was performed to estimate the degree of association between each dimension of occupational stress and the MBI-GS.

All data were analyzed using the Statistical Package for Social Sciences (version 21.0; SPSS Inc., Chicago, IL, USA).

### Ethics approval and Consent to participate

This study was approved by the Institutional Review Board of the Guangdong Province Hospital for Occupational Diseases Prevention and Treatment, and written consent was obtained from each participant.

## Results

### Participant characteristics

The present study comprised 1081 female workers. The average age of these participants was 30.85 ± 7.06 (18–54) years old. Approximately two-thirds (66.6%) of female workers were married, 139 (12.9%) of female workers were divorced, widows, or separated from their spouse, and 222 (20.5%) of female workers were single. Furthermore, over half of them (59.7%) had a junior high or low education level. Among all the respondents, 65.1% were shift workers, and 67.4% were exposed to occupational hazards. Other sociodemographic characteristics are listed in Table 1.

**Table 1:** Demographic and working characteristics of participants (*n* = 1081).

Variable	Number	Proportion (%)
**Age**		
< 25	254	23.5
25 – 30	299	27.7
30 – 35	253	23.4
> 35	275	25.4
**Marital status**		
Married	720	66.6
Divorced/Widow/Separated	139	12.9
Single	222	20.5
**Education level**		
Junior school or less	645	59.7
High school	360	33.3
Junior college or more	76	7.0
**Monthly income**		
< ¥1500	96	8.9
¥1500 ~	728	67.3
¥3000 ~	257	23.8
**Shift work**		
No	377	34.9
Yes	704	65.1
**Assembly line**		
No	264	24.4
Yes	817	75.6
**Working years (year)**		
< 2	427	39.5
2 ~ 4	268	24.8
> 4	386	35.7
**Exposure to occupational hazards**^a^		
No	352	32.6
Yes	729	67.4

^a^Occupational hazard is a hazard experienced in the workplace; it can encompass many types of hazards, including chemical hazards, biological hazards (biohazards), psychosocial hazards, and physical hazards.

### Comparison of scores in three dimensions of the MBI-GS in female workers

As shown in Table 2, it was found that age, marital status, educational level, monthly income, shift work, assembly line, working years and exposure to hazards all affected the emotional exhaustion score. The emotional exhaustion scores of female workers below 25 years old were higher than the scores of those in the other groups (*P* < 0.05). The emotional exhaustion scores of single female workers were higher than the scores of those in other groups (*P* < 0.05). The emotional exhaustion scores of female workers with a junior or lower educational level were higher than the scores of those in the other educational level groups *(P* < 0.05). The emotional exhaustion scores of female workers with a monthly income higher than ¥3000 were lower than the scores of those in the other groups (*P* < 0.05). The emotional exhaustion scores of female workers in the assembly line were higher than the scores of nonassembly line workers (*P* < 0.05). Compared with nonshift work female workers, shift work female workers had higher emotional exhaustion scores (*P* < 0.05). The scores of female workers exposed to occupational hazards were higher than those of female workers who were not exposed to occupational hazards (*P* < 0.05).

**Table 2:** Univariate analysis of MBI-GS scores according to demographics and work characteristics.

Variable	Number	Emotional exhaustion		Depersonalization		Personal accomplishment	
		Mean ± SE	*P* value	Mean ± SE	*P* value	Mean ± SE	*P* value
**Age**			< 0.01		0.282		< 0.01
< 25	254	12.80 ± 7.60		8.91 ± 7.06		20.85 ± 9.68	
25– 30	299	11.41 ± 7.63		8.37 ± 6.71		23.47 ± 9.46	
30– 35	253	10.49 ± 7.07		7.76 ± 6.16		24.20 ± 9.58	
> 35	275	10.53 ± 7.05		8.37 ± 6.44		23.27 ± 9.71	
**Marital status**			< 0.01		0.030		< 0.01
Married	720	10.82 ± 7.15		7.99 ± 6.37		23.57 ± 9.50	
Divorced/widow/separated	139	11.43 ± 7.63		9.39 ± 6.92		23.41 ± 9.50	
Single	222	12.74 ± 7.87		8.87 ± 7.09		20.79 ± 10.03	
**Education level**			< 0.01		< 0.01		< 0.01
Junior school or less	645	12.13 ± 7.71		8.85 ± 6.92		22.27 ± 9.87	
High school	360	10.04 ± 6.74		7.37 ± 5.83		24.18 ± 9.24	
Junior college or more	76	10.18 ± 6.78		8.74 ± 6.93		23.26 ± 9.35	
**Monthly Income**			< 0.01		< 0.01		< 0.01
< ¥1500	96	13.21 ± 8.71		9.82 ± 7.91		21.19 ± 11.51	
¥1500 ~	728	11.69 ± 7.41		8.59 ± 6.67		22.36 ± 9.59	
¥3000 ~	257	9.47 ± 6.45		7.13 ± 5.69		25.39 ± 8.70	
**Shift work**			< 0.01		< 0.01		0.771
No	377	10.00 ± 6.47		7.29 ± 5.89		23.09 ± 9.41	
Yes	704	11.99 ± 7.76		8.92 ± 6.90		22.91 ± 9.81	
**Assembly line**			< 0.01		0.229		< 0.01
No	264	9.94 ± 6.93		7.93 ± 6.31		24.44 ± 9.37	
Yes	817	11.74 ± 7.49		8.49 ± 6.70		22.50 ± 9.72	
**Working years (year)**			< 0.01		0.172		< 0.01
< 2	427	12.33 ± 7.96		8.81 ± 7.15		21.61 ± 10.00	
2 ~ 4	268	11.24 ± 7.14a		8.16 ± 6.22		23.79 ± 9.40	
> 4	386	10.19 ± 6.74		7.97 ± 6.23		23.92 ± 9.31	
**Exposure to occupational hazards**			< 0.01		0.380		0.806
No	352	10.36 ± 7.45		8.09 ± 6.77		22.87 ± 10.14	
Yes	729	11.75 ± 7.33		8.47 ± 6.53		23.02 ± 9.43	

In the dimension of depersonalization, the mean differences in marital status, educational level, monthly income, and shift work were statistically significant. The scores of female workers who were divorced/widowed/separated from their spouse were higher than the scores of married female workers (*P* < 0.05). Compared with other monthly income groups, subjects with a monthly income higher than ¥3000 had the lowest score (*P* < 0.05). Furthermore, the scores of shift female workers were higher than those of nonshift female workers.

For personal accomplishment, the mean differences were significant in terms of age, marital status, education level, monthly income, whether or not working in the assembly line, and working years. Female workers who were < 25 years old, single and in the assembly line and had < 2 practice years had lower personal accomplishment scores (*P* < 0.05). Furthermore, the scores of female workers with a monthly income of more than ¥3000 were higher than the scores of those in the other groups (*P* < 0.05).

### Correlation among variables of MBI-GS, JCQ and ERI

Table 3 lists the correlations among the MBI-GS, JCQ and ERI variables. Emotional exhaustion was positively correlated with depersonalization, job demand, effort, and overcommitment and negatively correlated with professional efficacy, social support, and reward. Depersonalization was positively correlated with emotional exhaustion, job demand, effort, and overcommitment and negatively correlated with professional efficacy and social support. Professional efficacy was positively correlated with job demand, social support, effort, and overcommitment and negatively correlated with emotional exhaustion and depersonalization. There was a lack of significant correlation between the MBI-GS and job control.

**Table 3:** Correlations among the dimensions of the MBI-GS, JCQ and ERI scores.

	1	2	3	4	5	6	7	8
1. Emotional exhaustion								
2. Depersonalization	0.662**							
3. Prefessional efficacy	− 0.164**	− 0.189**						
4. Job demand	0.274**	0.125**	0.125**					
5. Job control	− 0.047	0.020	0.020	− 0.053				
6. Social support	− 0.214**	0.164**	0.164**	0.077*	0.182**			
7. Effort	0.319**	0.119**	0.119**	0.495**	0.042	0.079**		
8. Reward	− 0.385**	0.010	0.010	− 0.375**	− 0.057	0.182**	− 0.478**	
9. Overcommitment	0.409**	0.082**	0.082**	0.378**	0.050	− 0.084**	0.502**	− 0.545**

*P < 0.05, **P < 0.01.

### The relationship between job burnout and occupational stress

The relationship between job burnout and occupational stress in the hierarchical linear regression analyses is presented in Table 4. The control variables (age, marital status, educational level, monthly income, shift work, assembly line, working years and exposure to occupational hazards) significantly predicted emotional (Δ*R*^2^ = 0.057), depersonalization (Δ*R*^2^ = 0.031) and professional efficacy (Δ*R*^2^ = 0.044) in step 1. Emotional exhaustion was positively associated with overcommitment, effort, and job demand and significantly reversely associated with social support and rewards. In the case of depersonalization, high levels of depersonalization were associated with high overcommitment and job demand, low social support and low rewards. Social support, job demand and rewards positively predicted professional efficacy. JDC and ERI were responsible for 23.5%, 19.6% and 4.6% of the variance in emotional exhaustion, depersonalization and professional efficacy, respectively.

**Table 4:** Hierarchical linear regression analysis of the factors associated with the MBI-GS scores.

Variables	Emotional exhaustion		Depersonalization		Professional efficacy	
	Step 1 (*β*)	Step 2 (*β*)	Step 1 (*β*)	Step 2 (*β*)	Step 1 (*β*)	Step 2 (*β*)
**Age**	− 0.066	− 0.064	− 0.001	− 0.008	0.021	− 0.004
**Marital status**						
Divorced/widow/separated	0.026	− 0.022	0.070*	0.025	− 0.007	− 0.004
Single	0.055	0.023	0.046	0.017	− 0.100**	− 0.084**
**Education level**						
High school	− 0.077*	− 0.047	− 0.048	− 0.021	0.100**	0.085**
Junior college or more	− 0.013	0.012	0.037	0.058	0.032	0.039
**Monthly income**						
¥1,500 ~	− 0.072	− 0.002	− 0.068	− 0.001	0.052	0.063
¥3,000 ~	− 0.103	− 0.030	− 0.129*	− 0.059	0.173**	0.179
**Shift work**	0.071	0.064*	0.068	0.050	0.081*	0.088*
**Assembly line**	0.049	0.037	0.022	0.011	− 0.043	-0.029
**Working years**	− 0.028	0.004	0.025	0.036	0.009	0.003
**Exposure to occupational hazards**	0.074*	0.018		− 0.020	0.032	0.023
**Job demand**		0.092*		0.068*		0.083**
**Job control**		− 0.039		0.001		0.005
**Social support**		− 0.175**		− 0.147**		0.137**
**Efforts**		0.100**		0.046		0.074
**Rewards**		− 0.148**		− 0.229**		0.097*
**Overcommitment**		0.230**		0.169**		0.073
R^2^	0.057**	0.292**	0.031**	0.226**	0.044**	0.090**
ΔR^2^	0.057**	0.235**	0.031**	0.196**	0.044**	0.046**

*P < 0.05, **P < 0.01.

The relationship between job burnout and occupational stress in the logistic regression is shown in Table 5. After adjusting for age, marital status, educational level, monthly income, shift work, assembly line, work age and occupational hazards, female workers exposed to high job strain were associated with high emotional exhaustion (OR = 2.27, 95% *CI*: 1.61–3.20) and high depersonalization (OR = 1.96, 95% *CI*: 1.45–2.64). A high degree of social support can reduce the risk of emotional exhaustion (OR = 0.37, 95% *CI*: 0.26–0.53) and depersonalization (OR = 0.49, 95% *CI*: 0.35–0.67), indicating that high social support is a mitigating factor of job burnout. Compared to female workers with low effort-reward imbalance, female workers with moderate and high effort-reward imbalance had a 2.45 times (95% *CI*: 1.66–3.61) and 2.88 times (95% *CI*: 1.89–4.38) risk of presenting emotional exhaustion, respectively. Compared to female workers with low effort-reward imbalance, female workers with high effort-reward imbalance had an increased risk of high depersonalization (OR = 1.96, 95% *CI*: 1.33–2.90). Moderate overcommitment can improve personal efficacy (OR = 1.52, 95% *CI*: 1.11–2.07), but high overcommitment can increase the risk of emotional exhaustion (OR = 3.07, 95% *CI*: 2.06–4.58) and depersonalization (OR = 2.83, 95% *CI*: 1.92–4.17).

**Table 5:** The ORs of job burnout by JCQ and ERI.

Variables	Emotional exhaustion^a^	Depersonalization^a^	Personal accomplishment^a^
	OR (95% *CI)*	OR (95% *CI*)	OR (95% *CI*)
**Job strain**			
Low	1	1	1
Moderate	1.37 (0.96–1.94)	1.39 (0.99–1.93)	1.04 (0.76–1.43)
High	2.27 (1.61–3.20)**	1.96 (1.45–2.64)**	1.21 (0.87–1.67)
**Social support**			
Low	1	1	1
Moderate	0.66 (0.48–0.92)*	0.83 (0.60–1.13)	1.32 (0.96–1.80)
High	0.37 (0.26–0.53)**	0.49 (0.35–0.67)**	1.75 (1.28–2.40)**
**Effort-reward imbalance**			
Low	1	1	1
Moderate	2.45 (1.66–3.61)**	1.40 (0.98–2.00)	1.06 (0.77–1.45)
High	2.88 (1.89–4.38)**	1.96 (1.33–2.90)**	1.13 (0.78–1.63)
**Overcommitment**			
Low	1	1	1
Moderate	1.45 (1.00–2.08)*	1.38 (0.98–1.96)	1.52 (1.11–2.07)**
High	3.07 (2.06–4.58)**	2.83 (1.92–4.17)**	1.44 (0.99–2.09)

*P < 0.05, **P < 0.01.

^a^Odds ratio adjusted by age, marital status, educational level, monthly income, shift work, assembly line, working years, and exposure to occupational hazards.

## Discussion

Occupational stress poses a threat to the health of workers and reduces the productivity of related organizations. The present study examined the relationship between occupational stress and job burnout in female manufacturing workers in Guangdong Province, China. These results support the hypothesis that job burnout is associated with occupational stress in female workers. Reducing occupational stress could be a useful intervention measure to control job burnout in female workers.

The present study found that sociodemographic characteristics, including age, marital status, educational level, monthly income, work shift, assembly line, working years and occupational hazard exposure, all had significant effects on the emotional exhaustion, depersonalization and personal accomplishment scores. Female workers who were < 25 years old and had < 2 practice years had higher levels of emotional exhaustion and lower levels of personal accomplishment, which was probably because they had just entered society, lacked work experience, had difficulties adapting to their work, and lacked the methods and skills to deal with work stress. Young people are usually not good at applying an effective network of interpersonal relationships in their work and lack a strong social support system. Being faced with heavy work pressure, they can neither cope with it effectively nor perform self-regulation, which is consistent with previous studies^26,27^. It is well known that support from a partner plays a major role in the face of adversity. Compared with married workers, single workers experience more emotional exhaustion and less personal accomplishment. Furthermore, educational level has significant effects on the emotional exhaustion, depersonalization, and personal accomplishment of female workers. Workers with lower educational levels have high levels of burnout. In Chinese belief, higher social status is usually associated with a higher level of education. As a result, female assembly line workers with low educational levels are more prone to job stress and burnout due to lack of self-confidence and heavy workload. Moreover, irregular work shifts could lead to circadian rhythm disruption, lifestyle changes, job strain and job burnout^28^. The present study found that female workers with shift work had higher emotional exhaustion and lower personal accomplishment than nonshift work female workers, which is consistent with the study conducted by Wang^28^. Female workers in manufacturing industries are exposed to multiple hazards, such as noise, chemical adhesive, and local vibration, which could be important contributors to mental health. The present study confirms that exposure to occupational hazards was related to job burnout.

A positive relationship between occupational stress and job burnout has been found in diverse occupational groups (migrant workers, doctors, and teachers)^18,19,28^. The present study revealed that occupational stress also plays an important role in burnout in female workers in electronic manufacturing industries.

Job demands and social support are closely correlated with emotional exhaustion and depersonalization. As the risk of emotional exhaustion and depersonalization increases with the increase in job strain, it decreases with the increase in social support. These correlations remained significant after adjusting for demographic and occupational characteristics, which is consistent with other reports^29,30^. Female manufacturing workers are almost completely occupied by machines, and they cannot control their work speed due to mechanized production. This suggests that high job strain is a risk factor that affects the job burnout of female workers, and social support is the mitigating factor of job burnout.

The model of effort-reward imbalance claims that there is a lack of reciprocity between costs and gains, such as a high cost-low gain condition provoking psychological distress^24^. Efforts are positively correlated with emotional exhaustion, while rewards are negatively correlated with emotional exhaustion and depersonalization. Female workers who experience high effort-reward imbalance are at higher risk of developing high emotional exhaustion and depersonalization. Overcommitment is regarded as a personality trait of cognitive, emotional, and motivational factors based on type A behavior, which reflects a need to be heavily recognized and respected. Individuals who are overcommitted will exaggerate their efforts due to their desire for esteem and approval and will find a discrepancy between efforts and reward to be particularly stressful^31^. The results of the present study show that overcommitment is positively correlated with emotional exhaustion and depersonalization. Hence, high overcommitment increases the risk of emotional exhaustion and depersonalization.

Several limitations should be acknowledged in our study. First, our study was a cross-sectional survey design, which means that assumptions about causality cannot be made. Second, we only selected large companies, which might limit the generalizability of our results. Third, data collection was self-reported, which could have a recall bias and makes it prone to social desirability. Face-to-face studies are recommended in the future to confirm our results. Further longitudinal studies as well as follow-up on the occupational stress and job burnout among workers exposed to occupational hazards would be suggested in the future.

## Conclusion

Taken together, the present study has a relatively large sample size. The present findings suggest that job burnout due to occupational stress presents an important perspective for industrial policy. Measures to reduce occupational stress are also an important way to prevent job burnout in female manufacturing workers in China. This cross-sectional study can only provide the present situation and the relevant relationship between occupational stress and job burnout. There is a lack of definitive evidence to guide the management of occupational stress and job burnout in manufacturing female workers. Determining how to alleviate occupational stress and job burnout requires further research. The investigators will continue to conduct research on intervention measures to provide reliable intervention measures for enterprises and relevant departments, creating a positive work climate and institutional support.

## Supplementary Information

Supplementary Table S1.

## Data Availability

The results and materials described in the article and the relevant raw data can be freely available upon request from the corresponding author.
