# Supplementary material for: The relationship between occupational stress and job burnout among female manufacturing workers in Guangdong, China: a cross-sectional study
Source: Sci Rep. 2022 Nov 23;12:20208. doi: 10.1038/s41598-022-24491-0 (PMC9684426; doi:10.1038/s41598-022-24491-0)
Supplement: Supplementary file 1 — Supplementary Table S1. [file 41598_2022_24491_MOESM1_ESM.docx]

**Table S1 The basic characteristics of 5 companies**

| Company | Economic type | Number of workers | Shift work | Average monthly salary |
| --- | --- | --- | --- | --- |
| A | Hong Kong, Macao and Taiwan capital | 500-800 | No | 2000-3000 |
| B | Foreign capital | ≥1500 | Yes | 3000-5000 |
| C | Foreign capital | <500 | Yes | 1000-2000 |
| D | Hong Kong, Macao and Taiwan capital | 500-800 | No | 2000-3000 |
| E | Hong Kong, Macao and Taiwan capital | 1000-1500 | No | 2000-3000 |
